# Atypical Role for PhoU in Mutagenic Break Repair under Stress in *Escherichia coli*

**DOI:** 10.1371/journal.pone.0123315

**Published:** 2015-05-11

**Authors:** Janet L. Gibson, Mary-Jane Lombardo, Ildiko Aponyi, Diana Vera Cruz, Mellanie P. Ray, Susan M. Rosenberg

**Affiliations:** 1 Department of Molecular and Human Genetics, Baylor College of Medicine, Houston, Texas, United States of America; 2 Department of Biochemistry and Molecular Biology, Baylor College of Medicine, Houston, Texas, United States of America; 3 Department of Molecular Virology and Microbiology, Baylor College of Medicine, Houston, Texas, United States of America; 4 Dan L Duncan Cancer Center, Baylor College of Medicine, Houston, Texas, United States of America; Louisiana State University and A & M College, UNITED STATES

## Abstract

Mechanisms of mutagenesis activated by stress responses drive pathogen/host adaptation, antibiotic and anti-fungal-drug resistance, and perhaps much of evolution generally. In *Escherichia coli*, repair of double-strand breaks (DSBs) by homologous recombination is high fidelity in unstressed cells, but switches to a mutagenic mode using error-prone DNA polymerases when the both the SOS and general (σ^S^) stress responses are activated. Additionally, the σ^E^ response promotes spontaneous DNA breakage that leads to mutagenic break repair (MBR). We identified the regulatory protein PhoU in a genetic screen for functions required for MBR. PhoU negatively regulates the phosphate-transport and utilization (Pho) regulon when phosphate is in excess, including the PstB and PstC subunits of the phosphate-specific ABC transporter PstSCAB. Here, we characterize the PhoU mutation-promoting role. First, some mutations that affect phosphate transport and Pho transcriptional regulation decrease mutagenesis. Second, the mutagenesis and regulon-expression phenotypes do not correspond, revealing an apparent new function(s) for PhoU. Third, the PhoU mutagenic role is not via activation of the σ^S^, SOS or σ^E^ responses, because mutations (or DSBs) that restore mutagenesis to cells defective in these stress responses do not restore mutagenesis to *phoU* cells. Fourth, the mutagenesis defect in *phoU*-mutant cells is partially restored by deletion of *arcA*, a gene normally repressed by PhoU, implying that a gene(s) repressed by ArcA promotes mutagenic break repair. The data show a new role for PhoU in regulation, and a new regulatory branch of the stress-response signaling web that activates mutagenic break repair in *E*. *coli*.

## Introduction

Bacterial, yeast and human cells generate mutations under growth-limiting stress by mutation mechanisms activated by stress responses [1–3]. These mechanisms increase genetic diversity, potentially accelerating adaptation, specifically when cells are maladapted to their environment. Although mutations can be deleterious, the adaptive value of mutagenesis under stress to bacterial populations is supported by mathematical modeling [4,5]. Various stress-induced mutagenesis mechanisms have been described in bacteria [1,2,6–8], yeast [9], and human cancer cells [3], the latter induced by hypoxic stress responses. Stress-induced mutagenesis mechanisms are induced in response to antibiotics [10,11] and anti-fungal drugs [12]. These various mechanisms produce base substitutions, and small insertions and deletions (indels), transpositions, gross chromosomal rearrangements, copy-number alterations and aneuploidy (above and [13]). Mechanisms of stress-induced mutagenesis are important to evolution of microbial pathogens (to drug resistance, increased virulence, escape of immune surveillance, for example) and also because of the mechanistic parallels to mutation in human cells, which pertain to oncogenesis, cancer progression, and resistance to chemotherapeutic agents [14].

In *Escherichia coli*, repair of DNA double-strand breaks by homologous recombination is non-mutagenic in unstressed cells, but under stress, switches to a mutagenic mode activated by stress responses [2,15,16]. Mutagenic break repair (MBR) is therefore a mechanism of stress-induced mutagenesis. MBR requires proteins that repair double-strand breaks (DSBs) via homologous recombination: RecA, RecBC and RuvABC, as well as error-prone DNA polymerase (Pol) IV (DinB), and to lesser extents Pols II and V, and activation of the SOS DNA-damage response, the σ^S^-controlled general/starvation stress-response, and the σ^E^ membrane-protein-stress response [2]. The membrane-protein-stress response promotes spontaneous DNA breakage in some DNA regions [17]. The SOS response is activated by DSBs and promotes mutagenesis by upregulation of DNA polymerases (Pols) IV and V. Break repair nevertheless remains non-mutagenic unless the σ^S^ response is also activated either by starvation (and presumably other inducers such as antibiotics [11]) or artificially. The general/σ^S^ stress response licenses the use of Pols IV, II and V in DSB repair by an as yet unknown mechanism, and thus executes the switch to mutagenic repair under stress in plasmids [15] and chromosomes of plasmid-free cells [16]. The MBR mechanism is supported by a 93-gene network, more than half of which functions in sensing stress and signal transduction that ultimately activates the σ^S^, σ^E^, and SOS responses, which allow mutagenesis [18].

**Fig 1:**
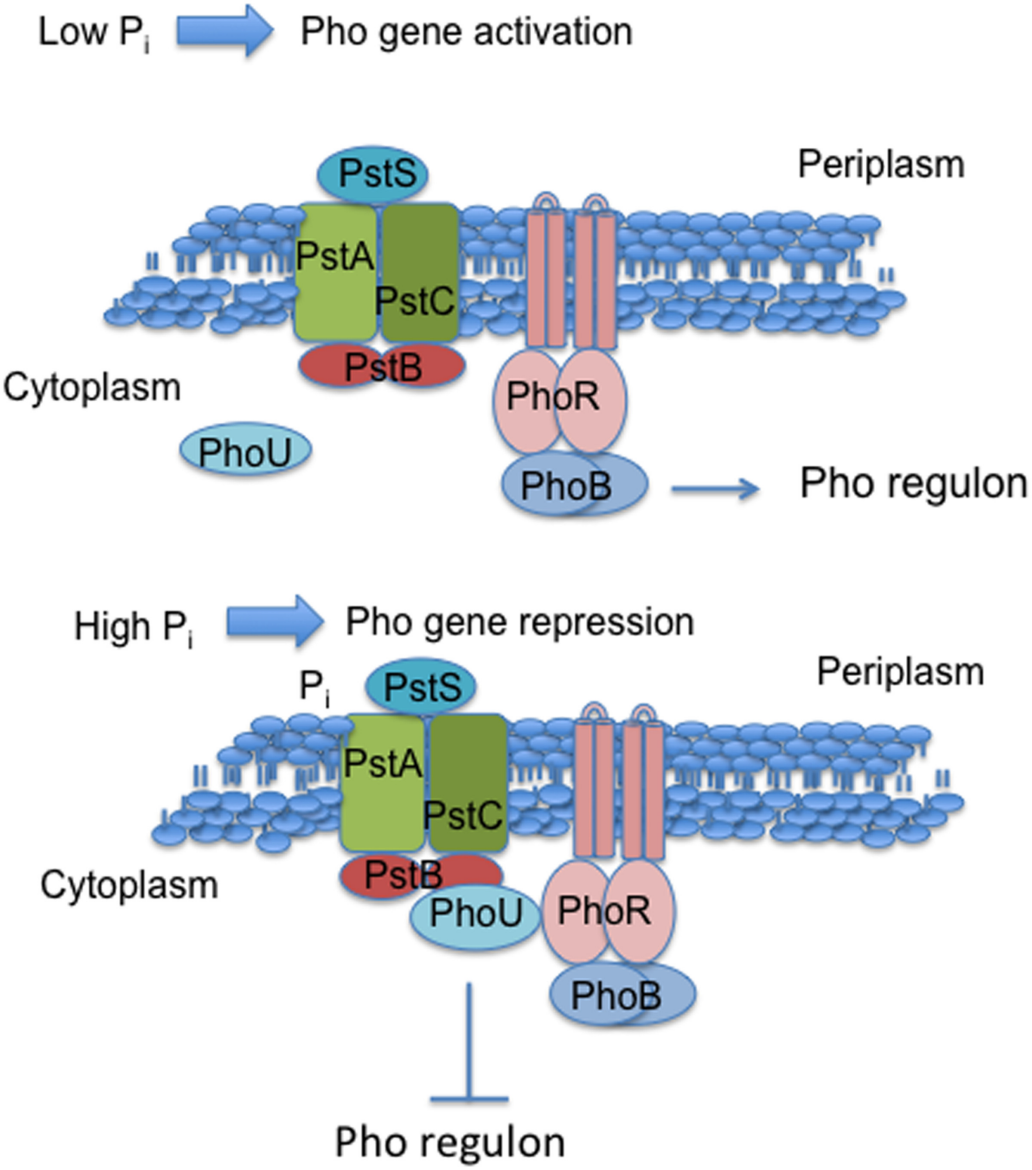
Model of regulation of the Pho regulon. Figure based on conclusions, models and interpretations of Hsieh and Wanner [19].

We developed a genetic screen for MBR-deficient mutants, screened transposon insertion libraries, and identified a network of 93 genes that contribute to MBR [18]. We focus here on mutations that affect PhoU, a regulator of the PhoBR phosphate utilization regulon. The PhoBR (hereafter termed Pho) regulon is induced in response to phosphate limitation, but also functions in transport of inorganic phosphate (P_i_) when P_i_ is in excess [19] and includes a large number of genes involved in transporting and catabolizing phosphate-containing compounds (reviewed by [20]) (Fig 1). PhoB, a response regulator, and PhoR, the membrane-bound sensor-kinase, comprise a two-component regulatory system that activates the Pho regulon, whereas PhoU is a poorly understood negative regulator of the Pho regulon. Genetic studies indicate that PhoB activity is repressed by the combined action of PhoR, PhoU, and the PstSCAB phosphate transport system in the presence of high extracellular phosphate concentrations [20] (Fig 1). Mutations in *phoU*, *phoR*, or *pstSCAB* result in de-repression of the regulon. These and other data have led to the proposal that the PstSCAB P_i_ transporter, PhoR, and PhoU, may form a chaperone-like signaling complex [19] at the membrane that inhibits PhoB-dependent transcription [20,21] (Fig 1).

Transposon insertions in *phoU* isolated in our screen cause a dramatic decrease in stress-induced MBR, and transposon insertions in *pstB* and *pstC* cause less severe but significant decreases in mutation, leading us to examine the role of the Pho regulon in stress-induced mutagenesis. We report that defects in phosphate transport and regulation can have both mild and dramatic effects on MBR uncorrelated with their known phosphate-regulatory roles.

## Materials and Methods

### Bacterial strains and growth conditions

Strains and plasmids used are listed in Table 1. Standard genetic techniques were used in strain construction [22]. All M9 minimal media [22] had carbon sources added at 0.1% and thiamine (vitamin B1) at 10 μg/ml. Antibiotic and other additives were used at the following final concentrations: chloramphenicol (Cam), 25 μg/ml; kanamycin (Kan), 50 μg/ml; tetracycline, 10 μg/ml; rifampicin, 100 μg/ml; 5-bromo-4-chloro-3-indolyl-phosphate-*p*-toluidine (XP), 4 μg/ml; 5-bromo-4-chloro-3-indolyl-β-D-galactoside (X-gal), 40 μg/ml; sodium citrate, 20 mM. The presence of *pst* and *pho* alleles that affect Pho-regulon expression were confirmed using Pho indicator plates [22] which contain the dye XP, a chromogenic substrate for alkaline phosphatase. Pho-de-repressed strains are dark blue on high-phosphate XP plates, whereas Pho-repressed strains are light blue to white [23].

**Table 1:** *Escherichia coli* strains and plasmids used.

Strain or plasmid	Relevant genotype	Reference or source
**ANCH1**	Δ(*phoB-phoR*)km^R^	[58]
**BW3904**	*phoU35*	[40]
**BW7150**	*ilvY864*::Tn*10*	B. Wanner
**BW13713**	*phoR68*(Oc)	[59]
**BW13989**	*proC*::Tn*5-132*	[23]
**BW17335**	Δ(*pstSCAB-phoU*)560::Kan	[23]
**CAG18599**	*ilvD3164*::Tn*10*Kan	*E*. *coli* Genetic Stock Center
**FC29**	Δ(*lac-proB*)_XIII_ *ara thi* [F´ *proAB* ^+^ Δ(*lacI-lacZ*)]	[32]
**FC40**	Δ(*lac-proB*) _XIII_ *ara thi* Rif^R^ [F´ *proAB* ^+^ *lacI33-lacZ*]	[32]
**SMR828**	Δ(*lac-proB*) _XIII_ *ara thi zaj-3053*::Tn*10*	[28]
**SMR3770** ^a^	FC40 [*F’ zah-*281::Tn*10 lacI33-lacZ*]^a^	[60]
**SMR3855-3856** ^b^, **SMR3858-3859** ^b^	FC40 Lac^+^ (day 5)	Independent isolates [41]
**SMR4045**	*ilvY864*::Tn*10 phoU35*	P1(BW7150) x BW3904
**SMR4047**	FC40 Δ(*pstSCAB-phoU*)*560*::Kan	P1(BW17335) x FC40
**SMR4056**	FC40 *ilvY864*::Tn*10 phoU35*	P1(SMR4045) x FC40
**SMR4058**	*proC*::Tn*5-132 phoR68*	P1(BW13989) x BW13713
**SMR4059**	FC40 *phoU35*	P1(FC40) x SMR4056
**SMR4060**	FC40 *proC*::Tn*5-132 phoR68*	P1(SMR4058) x FC40
**SMR4061**	FC40 *phoR68*	P1(FC40) x SMR4060
**SMR4562**	Δ(*lac-proB*) _XIII_ *ara thi* Rif^R^ [F´ *proAB* ^+^ *lacI33-lacZ*]	Independent construction of FC40 [44]
**SMR4604**	SMR4562 Δ(*phoB-phoR*)km^R^	P1(ANCH1) x SMR4562
**SMR4623**	SMR4562 *ilvD3164*::Tn*10*Kan	P1(CAG18599) x SMR4562
**SMR4953**	FC40 *phoU83*::Tn*10*dCam	This work, Results
**SMR4954**	FC40 *phoU84*::Tn*10*dCam	This work, Results
**SMR5235**	SMR4562 *phoU83*::Tn*10*dCam Δ(*phoB-phoR*)km^R^	P1(SMR4953) x SMR4604
**SMR5443**	SMR4562 Δ(*phoB-phoR*)km^R^ *proC*::Tn*5-132*	P1(BW13989) x SMR4604
**SMR5846**	SMR4562 Δ(*pstSCAB-phoU*)*560*::Kan Δ(*phoB-phoR*)km^R^ *proC*::Tn*5-132*	P1(SMR4047) x SMR5443
**SMR5860**	SMR4562 Δ(*pstSCAB-phoU*)*560*::Kan Δ(*phoB-phoR*)km^R^	P1(FC40) xSMR5846
**SMR6280**	FC40 Δ*araBAD567* Δ*attλ*::P_*BAD*_I-*SceI* [F' *mhpA32*::miniTn*7*Kan(I-*SceI* site)]	[15]
**SMR6281**	FC40 Δ*araBAD567* Δ*attλ*::P_*BAD*_ [F' *mhpA32*::miniTn*7*Kan(I-*SceI* site)]	[15]
**SMR6758**	SMR4562 Δ*pstS40*::Kan	SMR4562[pKD46] x short homology from pKD13 using primers ΔpstS1 and ΔpstS2
**SMR6759**	SMR4562 Δ*pstS41*	SMR6758 with Kan^R^ flipped out using pCP20
**SMR6760**	SMR4562 Δ*pstS41* Δ(*phoB-phoR*)km^R^	P1(ANCH1) x SMR6759
**SMR6761**	SMR4562 *phoU83*::Tn*10*dCam Δ*pstS40*::Kan	P1(SMR4953) x SMR6758
**SMR6762**	SMR4562 *phoU83*::Tn*10*dCam Δ*pstS41*	SMR6761 with Kan^R^ flipped out using pCP20
**SMR7351**	SMR4562 *phoU83*::Tn*10*dCam Δ*pstS41* Δ(*phoB-phoR*)km^R^	P1(ANCH1) x SMR6762
**SMR10308**	SMR4562 [F′ *lafU2*∷FRTcatFRT *dinBo-21*(o^c^)]	[45]
**SMR10865**	FC36 Δ*araBAD*567 Δ*zie3913*.*1*::*tetRtetA+1*FRT Δ*zie3920*.*5*::3ChiKanISceIsite	[16]
**SMR10866**	FC36 Δ*araBAD*567 Δ*attλ*::P_*BAD*_I-*Sce*I Δ*zie3913*.*1*::*tetRtetA+1*FRT Δ*zie3920*.*5*::3ChiKanISceIsite	[16]
**SMR12566**	SMR4562 *rssB*::Tet	[18]
**SMR12672**	SMR4562 Δ*arcA726*::FRT	[18]
**SMR12673**	SMR4562 Δ*arcB738*::FRT	[18]
**SMR13353**	SMR4562 Δ*phoU*::FRTKan	(17)
**SMR17049**	SMR4562 [F′ *lafU2*∷FRT *dinBo-21* (o^c^)]	SMR10308 x pCP20
**SMR19235**	FC40 Δ*araBAD567* Δ*attλ*::P_*BAD*_I-*SceI phoU83*::Tn*10*dCam [F' *mhpA32*::miniTn*7*Kan(I-*SceI* site)]	P1(SMR4953) x SMR6280
**SMR19236**	FC40 Δ*araBAD567* Δ*attλ*::P_*BAD*_ *phoU83*::Tn*10*dCam [F'*mhpA32*::miniTn*7*Kan(I-*SceI* site)]	P1(SMR4953) x SMR6281
**SMR19248**	SMR4562 *rssB*:: tet *phoU83*::Tn*10*dCam	P1(SMR4953) x SMR12566
**SMR19249**	SMR4562 *arcA726*::FRTKan *phoU83*::Tn*10*dCam	P1(SMR4953) x SMR12672
**SMR19250**	SMR4562 *arcB738*::FRTKan *phoU83*::Tn*10*dCam	P1(SMR4953) x SMR12673
**SMR20214**	SMR4562 *phoU83*::Tn*10*dCam [F′ *lafU2*∷FRT *dinBo-21*(o^c^)]	P1(SMR4953) x SMR17049
**SMR20344**	FC36 Δ*araBAD*567 Δ*attλ*::P_*BAD*_I-*Sce*I Δ*zie3913*.*1*::*tetRtetA+1*FRT Δ*zie3920*.*5*::3ChiKanISceIsite *phoU83*::Tn*10*dCam	P1(SMR4953) x SMR10866
**SMR21643**	FC40 Δ*araBAD567* Δ*attλ*::P_*BAD*_I-*SceI* [F' *mhpA32*::miniTn*7*Kan(I-*SceI* site)] *phoU83*::Tn*10*dCam *pstB*	P1(SMR4953) x SMR6281
**SMR21644**	FC36 Δ*araBAD*567 Δ*attλ*::P_*BAD*_I-*Sce*I Δ*zie3913*.*1*::*tetRtetA+1*FRT Δ*zie3920*.*5*::3ChiKanISceIsite *phoU83*::Tn*10*dCam *pstB*	P1(SMR4953) x SMR10866
**pKD46**	*ori101 repA101ts* P*BAD*-*gam-bet-exo* AmpR	[24]
**pKD13**	Source of FRT*cat*FRT	[24]
**pCP20**	Yeast Flp recombinase on a temperature-sensitive replicon λ*pr*-*FLP*, λ*c*I*ts857*, RepTS, AmpR, CamR	[25]

^a^This strain is derived from a Lac^+^ colony isolated from a stress-induced mutagenic break-repair experiment and so may carry additional mutations.

^b^These are independent Lac^+^ stress-induced point mutants. See [61], for the sequence to which the nt positions correspond.

A *pstS* nonpolar deletion allele, Δ*pstS41* was constructed using short homology recombineering [24] using primers ΔpstS1 (5’GCTTTATGAATCCTCCCAGGAGACATTATGAAAGTTATGATTCCGGGGATCCGTCGACC) and ΔpstS2 (5’ACACCGTACCCGGCCTGGAGTTTTATTAGTACAGCGGCTTTGTAGGCTGGAGCTGCTTC) and pKD13 as template to create Δ*pstS40*::Kan. Δ*pstS41* (created by removal of Kan from Δ*pstS40*::Kan per [25]) caused de-repression of the Pho regulon, observed on XP medium, as expected. Bases 12–1035 of *pst* are deleted in Δ*pstS40* and Δ*pstS41*.

### Stress-induced mutagenic break-repair assays

Stress-induced Lac-reversion assays were performed as described [26] at 37°C. *phoU83*::Tn*10*dCam strains were concentrated 10-fold before plating to obtain sufficient Lac^+^ colony counts. All experiments presented showed less than two-fold net population change during the first 1–3 days after plating per [26]. Stress-induced mutation assays with I-*Sce*I-produced DSBs were performed exactly as described [15]. The chromosomal *tet* +1bp frameshift assay was carried out as described [16] using the *tet2* allele and I-site A [16].

### Reconstruction experiments

Reconstruction experiments to determine the speed of colony formation of Lac^+^ derivatives of various mutants under exact selective experimental conditions, in the presence of neighbor (scavenger) cells, which consume any non-lactose carbon sources present, were performed as described [27].

### Generation-dependent mutation-rate determinations

Fluctuation tests were used to determine frequencies of generation-dependent Lac^+^ revertants formed in rapidly growing cells as described previously [28,29]. Mutation rates were estimated from these mutant frequencies based on a modified method of the median [30,31]. To determine Lac^+^ mutant frequencies, rather than scoring only at 48 hr, we plated several independent Lac^+^ derivatives of each strain in parallel and scored all strains for Lac^+^ colonies several times over a 4–6 hr period (see Results for rationale for this approach, and reviewed by [29]). Lac^+^ derivatives were confirmed to be stably Lac^+^ rather than unstably Lac^+^, due to amplification of the leaky *lac* allele, by scoring Lac^+^ phenotypes on rich medium containing X-gal [27]. For each genotype, a t_50_ to colony formation (time at which 50% of the Lac^+^ control colonies were visible) was calculated and the median Lac^+^ mutant frequency at the t_50_ was used to calculate the mutation rate to Lac^+^. A final cell count was taken after 4 to 5 days when no further Lac^+^ control colonies were appearing (t_100_) and used to calculate the t_50_. The mutation rates were then multiplied by two to give the rate at t_100_.

### Whole-genome sequencing

Genomic DNA was extracted from each strain and purified for sequencing using DNeasy Blood & tissue kits (Qiagen). Sequencing was performed in a Mi-Seq using Nextera XT kits for library preparation, producing paired-end reads of ~150 nt. The data were processed using CASAVA 1.8a5 software; the reference genome was MG1655 (NCBI accession number: NC_000913.3) corrected for the 81 SNVs present in SMR4562 (the Lac-assay strain) discovered by our laboratory, and the sequence of plasmid F’128 retrieved from http://rothlab.ucdavis.edu/refseqs/fc40.fasta. Apparent variants (mutations, or SNVs) were filtered such that those present in ≥70% of reads of any segment containing the variant were called as variants. A subsequent alignment of the reads using BLASTn was made to detect reads that contained noncontiguous sequences in the reference genome, and so confirm indels and detect boundaries of possible genome rearrangements. In the genome sequences reported, there were no indels or genome-rearrangement junctions detected.

## Results

### The Lac MBR assay

**Fig 2:**
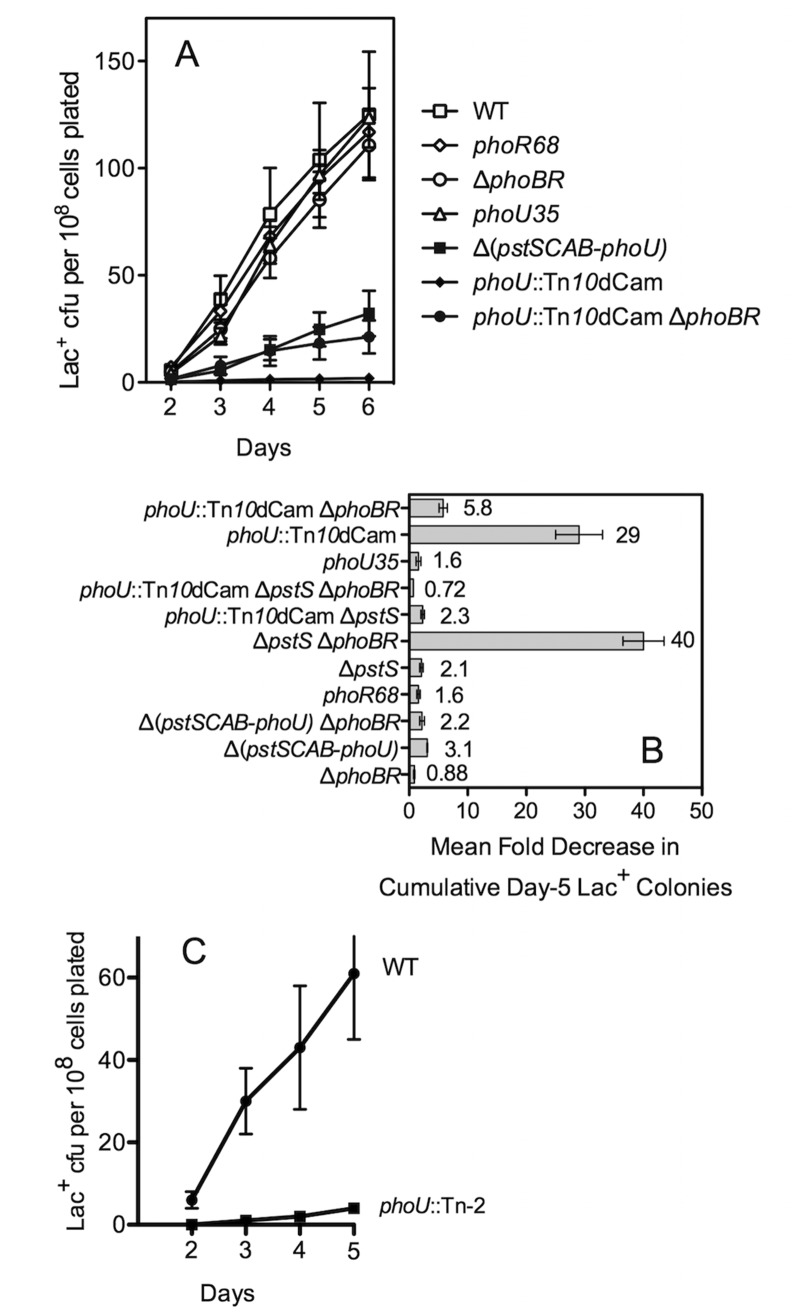
Mutations affecting the Pho regulon can decrease stress-induced Lac^+^ reversion. (A) Representative experiment. Strains (top to bottom in legend): SMR4562, SMR4061, SMR4604, SMR4059, SMR4047, SMR4953, and SMR5235. Values are means ± one SEM for eight independent cultures per strain in one representative experiment (where not visible, error bars are smaller than the symbol). (B) Mean of multiple experiments. Complex effects of double and triple mutations affecting the Pho regulon on MBR in the Lac assay. Strains (top to bottom in legend): SMR5235, SMR4953, SMR4059, SMR7351, SMR6762, SMR6760, SMR6759, SMR4061, SMR5860, SMR4047, and SMR4604. Fold decrease in the change in Lac^+^ from day 4 to day 5 relative to the *pho* ^+^ strain SMR4562 was calculated for each genotype in several experiments of multiple cultures (like that shown in A). The values (shown next to the bars) are the mean fold decreases in mutagenesis from multiple experiments ± SEM (error bars, n ≥ 3). (C) A different *phoU*::Tn*10* transposon insertion (SMR4954) also depresses MBR, indicating that the *phoU* mutagenesis-deficiency is not the result of a specific truncation/fusion protein. Representative experiment.

Mutagenic break repair (MBR) can be observed using the *E*. *coli* Lac assay [32], and several other assay systems (e.g., [16,18]), some used here. In the Lac assay cells with a *lac* +1bp frameshift mutation in an F’ conjugative plasmid are starved on solid minimal medium with lactose as the sole carbon source. Lac^+^ revertant colonies accumulate from the second day after plating onward for more than seven days (e.g., Fig 2A). The majority of Lac^+^ colonies that appear before day 6 carry a compensatory frameshift “point” mutation [33,34]; a minority carry an amplified array of tandem copies of the leaky *lac* allele which confers enough β-galactosidase activity to allow growth [27]. Here we use this (and other) assays, and score the Lac^+^ colonies before day 6, and so focus principally on the point-mutagenesis MBR mechanism which generates base substitutions and indels [2].

Mutagenesis in this assay is known to result from mutagenic repair of spontaneous DNA double-strand breaks (DSBs) near *lac* as follows. The specific mutagenic pathway acting, which requires the DSB-repair proteins, error-prone DNA Pols and stress-response proteins reviewed above—acts only in DNA molecules that have DSBs [15,16]; forms mutations near the DSB site [35]; is inhibited by a phage DSB-end trapping protein that blocks repair [36]; and requires RecBC, a specific DSB-binding repair protein [37].

### *phoU* mutations inhibit mutagenic break repair

In a general screen for MBR-deficiency using the *E*. *coli* Lac assay and transposon mutagenesis, two unpublished transposon insertions in *phoU* were isolated in an earlier version of the screen of [18]. Both of the insertions are near the C-terminal end of the *phoU* coding region. The *phoU* C-terminal insertions including *phoU83*::Tn*10*dCam have severe mutagenesis defects [18] (Fig 2A–2C). Two additional mutations isolated in genes *pstA* and *pstC*, which encode part of the phosphate-specific ABC transporter, PstSCAB, exhibit relatively modest mutagenesis defects [18]. A second assay for MBR measures indel mutation via reversion of a chromosomal *tet* frameshift reporter gene in plasmid-free cells to which a DSB has been delivered near the reporter gene using a regulatable I-*Sce*I endonuclease [16]. We show below that *phoU83*::Tn*10*dCam cells are defective in MBR in this chromosomal assay as well.

The insertion in SMR4953 (*phoU83*::Tn*10*dCam, used throughout this work) has Tn*10*dCam inserted such that the last 23 amino acids of the predicted 241-amino-acid PhoU protein are replaced with 12 amino acids encoded by the transposon (ADESPNDFGKNH). This insertion reduced MBR in the Lac assay dramatically (Fig 2A, closed diamonds). Similarly reduced mutagenesis was seen with a different insertion (Fig 2C) in which the last 21 amino acids of PhoU are replaced. A third *phoU* transposon insertion in the ribosome-binding site also showed reduced MBR [18]. Thus, the reduction in mutagenesis is not the result of a specific fusion protein. The data below indicate that a function of wild-type PhoU is required for MBR.

### *phoU83*::Tn*10*dCam is a separation-of-function allele that retains the PhoU essential function

We show that the *phoU83*::Tn*10*dCam allele is a partial-loss-of-function allele in that it is deficient in Pho regulon repression but retains the PhoU essential function as follows. First, cells carrying *phoU83*::Tn*10*dCam are unable to repress the Pho regulon, as seen by their blue-colony phenotype on XP phosphate-indicator medium (Table 2) indicating de-repression of the Pho regulon (Materials and Methods) [23]. Second, further indicating reduced PhoU function, *phoU83*::Tn*10*dCam causes a slow-growth (small-colony) phenotype (Table 2). However, third, *phoU83*::Tn*10*dCam is demonstrably *not* a null allele as follows. PhoU is an essential gene, though the nature of its essential function is unknown [23]. Null mutants are viable only with an additional suppressor mutation in any of the *pstSCAB* or *phoBR* genes [23]. These suppressor mutations cause rapid growth and large colonies [23]. We find that *phoU83*::Tn*10*dCam strains show a stable small-colony phenotype (Table 2), and do not carry suppressor mutations as shown by whole-genome sequencing (unless grown rapidly on glucose medium, discussed below), and so are not *phoU* null mutants. Two *phoU83*::Tn*10*dCam (small-colony) strains were sequenced, SMR20344 and SMR4953, and neither contained mutations in the *pstSCAB* or *phoBR* genes indicative of suppression [23]. The first contained no mutation in its genome (relative to its isogenic parent) other than *phoU83*::Tn*10*dCam. SMR4953 carries an apparently incidental silent *bioA* mutation encoding a W186L substitution that does not cause auxotrophy. As a positive control, when grown rapidly on glucose medium, *phoU83*::Tn*10*dCam strains derived from SMR6280 and SMR10866 acquired large-colony phenotype and carried suppressor mutations in *pstB* (Table 2). We conclude that *phoU83*::Tn*10*dCam is a separation-of-function allele that retains the PhoU essential function but is defective in Pho-regulon repression.

**Table 2:** Pho-regulon-repression defect without *pst/phoBR* suppressor mutations in *phoU83*::*Tn*10dCam strains.

Strain	Pho genotype	Colony size	Colony color on phosphate indicator	*pst* or *phoBR* mutations
**SMR4562**	*phoU* ^+^	Large	White	none
**SMR4953**	*phoU83*::*Tn*10dCam	Small	Blue	none
**SMR13353**	Δ*phoU*::FRTKan	Large	Blue	*pstB* 7bp deletion after Y256
**SMR20344**	*phoU83*::*Tn*10dCam	Small	Blue	none
**SMR21643**	*phoU83*::*Tn*10dCam	Large	Blue	*pstB* 2bp deletion after aa153
**SMR21644**	*phoU83*::*Tn*10dCam	Large	Blue	*pstB* P14S

Colony size was observed on M9 B1 glycerol medium. *pstSCAB* suppressor mutations or their absence were identified either by whole-genome sequencing (strains SMR4562, SMR4953, SMR20344, SMR21643, SMR21644) or by targeted sequencing of those genes (SMR13353).

### Neither poor growth nor de-repression of the Pho regulon account for whole mutagenesis deficiency

We tested whether the *phoU* mutagenesis deficiency might relate to the growth defect known to be associated with increased PstSCAB protein function in *phoU* strains (in which PhoU cannot inhibit the PstSCAB growth-inhibitory activity [38,39]), which presumably accounts for the small-colony morphology of *phoU83*::Tn*10*dCam cells. We did this by examining *phoU* strains that carry mutations that decrease PstSCAB function, and so should have no growth defect. We did this by reducing PstSCAB function in several different ways. We created Δ*pstS*, a nonpolar in-frame deletion (Materials and Methods). In a Δ(*pstSCAB-phoU*) strain and a *phoU*::Tn*10*dCam Δ*pstS* strain, PstSCAB function is eliminated, and in a *phoU*::Tn*10*dCam Δ*phoBR* strain, lacking its transcriptional activator, PstS-dependent PstSCAB function is low, remaining at uninduced levels [23]. These reductions in PstSCAB activity allow large-colony formation (not shown) but do not restore mutagenesis proficiency. The Δ(*pstSCAB-phoU*), *phoU*::Tn*10*dCam Δ*pstS* and *phoU*::Tn*10*dCam Δ*phoBR* strains form normal-size colonies, yet still have somewhat reduced MBR (Fig 2A and 2B) indicating that increased PstSCAB and consequent growth defect is not the sole cause of the mutagenesis defect in *phoU83*::Tn*10*dCam cells. The Δ(*pstSCAB-phoU*) and *phoU83*::Tn*10*dCam Δ*pstS* strains (with no PstSCAB activity) manifest three- and two-fold decreases in mutant-colony production, respectively (Fig 2A and 2B). This is significantly less mutagenesis than in *pho* ^+^ (*P* = 0.027 and 3.2 × 10^-5^, respectively, t-test) but significantly more mutagenesis than in *phoU83*::Tn*10*dCam (Fig 2A and 2B), and implies that the strong mutagenesis defect of *phoU83*::Tn*10*dCam may relate partially but not solely to increased PstSCAB activity. Suggesting that blocking induction of the Pho regulon, including *pstSCAB*, only partially alleviates the *phoU83*::Tn*10*dCam phenotype, we see that mutagenesis in the *phoU83*::Tn*10*dCam Δ*phoBR* (*pstSCAB-*uninducible) strain is reduced about five-fold relative to *pho* ^+^ (Fig 2A and 2B, *P* = 1.2 × 10^-5^, t-test). All of these results imply that in wild-type PhoU^+^ cells, repression of PstSCAB promotes MBR. However, the less severe mutation-down phenotype in *phoU83*::Tn*10*dCam strains that lack PstSCAB implies that an additional function of PhoU may exist outside of repression of the Pho operon and promotes MBR.

### De-repression of the Pho regulon does not correlate with MBR deficiency

De-repression of the Pho regulon is not required for mutagenesis because the Δ*phoBR* strain, in which the gene encoding the transcriptional activator PhoB is deleted, has mutation levels similar to *pho* ^+^ (Fig 2A and 2B). This is not surprising because the Pho regulon is repressed on the high phosphate M9 medium on which the mutagenesis experiments are performed. However, perhaps the inappropriate de-repression of the regulon associated with *pstSCAB* or *phoU* mutations [20] is mildly detrimental to mutagenesis. We tested this in several ways.

First, indicating that Pho regulon de-repression *per se* does not inhibit mutagenesis, we find that *phoU35*, a mutant with de-repressed Pho regulon but rapid growth (large colonies without suppressor mutations) has normal mutagenesis. *phoU35* is an altered-function allele [40], which causes de-repression of the Pho regulon in high phosphate conditions, but does not cause the slow-growth phenotype associated with a *phoU* deletion. These phenotypes suggest that the PhoU35 protein retains the ability to block the PstSCAB growth-inhibitory function, but cannot mediate repression of the Pho regulon (reviewed, [20]). We find that *phoU35* does not affect mutagenesis (Fig 2A and 2B), suggesting that the mutagenesis defect of *phoU83*::Tn*10*dCam is not caused by simple de-repression of the Pho regulon.

Second, we find that the *phoR68* null allele, which also leads to regulon de-repression [20], also had no effect on MBR (Fig 2A and 2B).

Third, loss of PstSCAB function achieved *via* the null alleles Δ*pstS* or Δ(*pstSCAB-phoU*), both of which also cause de-repression (reviewed, [20], Materials and Methods), decrease mutagenesis roughly two- and three-fold, respectively (Fig 2A and 2B, *P =* 1.4 × 10^-8^ and -2.0 × 10^-8^, t-test), not the >20-fold of *phoU*::Tn*10d*Cam (Fig 2A and 2B) or *phoU*::Tn-2 (Fig 2C). We show that de-repression does not account for the MBR reduction in Δ(*pstSCAB-phoU*) by showing that combining Δ(*pstSCAB-phoU*) with the Δ*phoBR* allele, which makes the Pho regulon uninducible, does not alter the mutagenesis defect: Δ(*pstSCAB-phoU*) Δ*phoBR* is as MBR-deficient as Δ(*pstSCAB-phoU*) cells (Fig 2B, *P =* 0.5018, t test).

Unexpectedly, introducing the Δ*phoBR* allele into Δ*pstS* cells to create the Δ*pstS* Δ*phoBR* strain (*phoU* ^+^) strain reduced mutagenesis >40-fold, a stronger phenotype than that of *phoU83*::Tn*10*dCam (Fig 2B). Again, because the Pho regulon is uninducible in this strain because of the Δ*phoBR* mutation, this MBR-deficiency is not caused by Pho-regulon de-repression. The Δ*pstS* Δ*phoBR* strain does not have the small-colony phenotype of the *phoU83*::Tn*10*dCam, implying a different block to mutagenesis—not growth inhibition. The strong Δ*pstS* Δ*phoBR* mutagenesis defect contrasts with that of two Δ*phoBR* strains that lack *phoU* ^+^: the Δ(*pstSCAB-phoU*) Δ*phoBR* strain, in which mutagenesis is decreased only 2-fold, and *phoU83*::Tn*10*dCam Δ*phoBR*, in which mutagenesis is reduced 6-fold (Fig 2B). These data imply that *phoU* ^+^ may be responsible for the strong reduction in mutagenesis in the absence of *pstS* and *phoBR*. To explore this further we constructed the triple mutant strain *phoU83*::Tn*10*dCam Δ*pstS* Δ*phoBR*, and found that it has wild-type levels of mutagenesis (Fig 2B). Thus, it appears that either *pstS* ^+^ or *phoBR* ^+^ is required for mutagenesis in a *phoU* ^+^ background and that either *pstS* or *phoBR* must be absent for mutation to occur in a *phoU*-mutant background. The lack of correlation between de-repression of the Pho regulon and MBR phenotypes in the experiments above rules out a role for simple Pho regulon de-repression and suggests some other role of PhoU in mutagenesis. The unexpected phenotypes in PhoU^+^ strains suggest multiple routes for Pho regulation and regulators to impact MBR and roles for PhoU other than simple modulation of repression and constraining PstSCAB function.

### Slow Lac^+^ colony formation in *pho* strains does not account for MBR defects

We used reconstruction experiments to show that poor colony formation under selective conditions is not the major cause of MBR-deficiency of *pho* strains. In reconstruction experiments, a known number of Lac^+^ cells are plated under the conditions of a stress-induced-mutation experiment: on lactose minimal medium with neighbor cells present (Δ*lac* cells that scavenge any non-lactose carbon sources, reviewed [41,42]). Most day-5-Lac^+^ mutant colonies are visible as colonies two days after plating [41]. We find that the two strains with the strongest mutagenesis defects (*phoU83*::Tn*10*dCam and Δ*pstS* Δ*phoBR*), and none of the other strains, show altered growth in these reconstruction experiments. The *phoU83*::Tn*10*dCam strain forms colonies slightly more slowly than its *pho* ^+^ parental strain (three days *versus* two, Table 3), but with poor efficiency; only about 15–20% of the *phoU83*::Tn*10*dCam Lac^+^ cells ultimately form colonies (Table 3). This poor efficiency of colony formation is not seen when *phoU83*::Tn*10*dCam Lac^+^ cells are plated on selective medium without scavenging neighbor cells, suggesting poor competition with the faster growing *pho* ^+^ scavenger cells (data not shown). The Δ*pstS* Δ*phoBR* strain formed colonies efficiently but slightly more slowly than its isogenic parents (about three days) and variably from culture to culture (Table 3). This suggests, first, that the few late Lac^+^ mutants generated in the mutation-deficient Δ*pstS* Δ*phoBR* strain (Fig 2B) might include slow-growing generation-dependent mutants such that the strain might be more deficient in MBR than is evident. Second, this average one-day lag in colony growth cannot account for the strong mutagenesis defect of this strain. We conclude that at least some of the effects of *phoU83*::Tn*10*dCam on Lac reversion may be *via* poor colony formation under selective conditions. The MBR defect of *phoU83*::Tn*10*dCam is confirmed in a separate chromosomal assay not subject to these concerns, below, further demonstrating roles of in mutagenesis independent of colony growth rates.

**Table 3:** *pho* mutation effects on speed and efficiency of Lac^+^-colony formation do not account for MBR-deficiency.

Strain	Relevant genotype	Average days to Lac^+^ colony formation^a^		% viable cells forming Lac^+^ colonies^a^	
		Exp. 1	Exp. 2	Exp. 1	Exp. 2
**SMR4562**	*pho* ^+^	2.1 ± 0.03	2.0 ± 0.02	100 ± 7	110 ± 10
**SMR4059**	*phoU35*	2.2 ± 0.2	2.0 ± 0.3	81 ± 11	96 ± 10
**SMR4061**	*phoR*	2.7 ± 1	2.5 ± 0.8	100 ± 20	91 ± 20
**SMR4953**	*phoU*::Tn*10*dCam	3.4 ± 0.74	2.5 ± 1.2^b^	18 ± 12	13 ± 7
**SMR4047**	Δ(*pstSCAB-phoU*)	2.2 ± 0.35	2.1 ± 0.15	92 ± 9	87 ± 36
**SMR6759**	Δ*pstS*	2.2 ± 0.32	2.1 ± 0.15	93 ± 10	97 ± 22
**SMR4604**	Δ*phoBR*	2.1 ± 0.03	2.0 ± 0.02	100 ± 8	100 ± 15
**SMR6762**	*phoU*::Tn*10*dCam Δ*pstS*	2.6 ± 0.5	2.3 ± 0.39	96 ± 9	95 ± 40
**SMR5860**	Δ(*pstSCAB-phoU)* Δ*phoBR*	2.5 ± 0.53	2.2 ± 0.25	98 ± 13	105 ± 30
**SMR6760** ^c^	Δ*pstS* Δ*phoBR*	3.2 ± 1.0	3.1 ± 0.93	91 ± 8	92 ± 13

^a^Values are means ± one standard deviation (SD). In each case, four-six independent day-5 or day-6 Lac^+^ mutants were used as controls for the time of colony formation (see Materials and Methods), with the exception of SMR4562 for which day-2 mutants were used (they behave similarly [41]). Exp. 1 was carried out to day 5, and Exp. 2 to day 6.

^b^ Only two Lac^+^ control strains were used in this case and so a range, rather than SD is given.

^c^ We note that the time to Lac^+^ colony formation for different isolates of Δ*pstS* Δ*phoBR* strain SMR6760 varies from two to five days. They all form normal-size colonies and are not detectably amplified (amplified Lac^+^ take 3–5 days to form, [27]).

### Weak effects of *pho* mutations on generation-dependent Lac^+^ reversion in rapidly growing cells are uncorrelated with MBR defects

Generation-dependent Lac^+^ reversion in rapidly growing cells occurs by multiple molecular mechanisms other than, and in addition to, MBR [1,2,16]. We examined the generation-dependent mutation rates to Lac^+^ to determine whether *pho* mutations also affect mutagenesis mechanisms other than MBR, which is confined to stationary-phase and other times of σ^S^-response induction [2,15,16]. An equalizing method was used that minimizes the possible inclusion of stress-induced mutants and accounts for possibly different times-to-Lac^+^-colony formation for different genotypes [29,43] (see Materials and Methods). For example, because the Δ*pstS* Δ*phoBR* strain forms Lac^+^ colonies more slowly than the wild-type (Table 3), we score generation-dependent Lac^+^ revertant colonies of this strain proportionately later than for the wild-type. We were unable to determine a generation-dependent Lac^+^ reversion rate for the *phoU83*::Tn*10*dCam strain due to poor efficiency of colony formation by Lac^+^ cells under the selective conditions (see above, Table 3), but all other *pho* mutants (Table 4) have only modest 2-4-fold effects on generation-dependent Lac^+^ reversion in growing cells. Due to the nature of mutation rate determinations, more experiments would be required to conclude that these small differences were significant. The lack of correlation between these rates and the phenotypes in stress-induced Lac^+^ reversion implies that the effects are specific to stress-induced MBR. Certainly the Δ*pstS* Δ*phoBR* combination affects stress-induced Lac^+^ reversion more strongly (40-fold) than generation-dependent reversion (4-fold, Table 4).

**Table 4:** *pho* mutations do not strongly affect generation-dependent Lac^+^ reversion rates.

Strain	Relevant genotype	Mutation rate x 10^-9^ (mutations/cell/generation)^a^		
		Exp. 1	Exp. 2	Avg.
**SMR4562**	*pho* ^+^	3.6	3.7	3.7
**SMR4047**	Δ(*pstSCAB-phoU*)	4.2	12	8.1
**SMR6759**	Δ*pstS*	4.8	4.7	4.8
**SMR4604**	Δ*phoBR*	4.0	1.4	2.7
**SMR6760**	Δ*pstS* Δ*phoBR*	1.0	0.99	1.0
**SMR5235**	*phoU*::Tn*10*dCam Δ*phoBR*	1.4	0.79	1.1

^a^Mutation rates were calculated as described in Materials and Methods. Exp. 1 and 2 consisted of 19 and 14–15 independent cultures of each strain respectively. Four to six Lac^+^ derivatives of each strain were plated in parallel as controls as described in Materials and Methods.

### Neither I-*Sce*I-induced DSBs nor SOS-induced levels of DinB substitute for PhoU^+^ in mutagenesis

**Fig 3:**
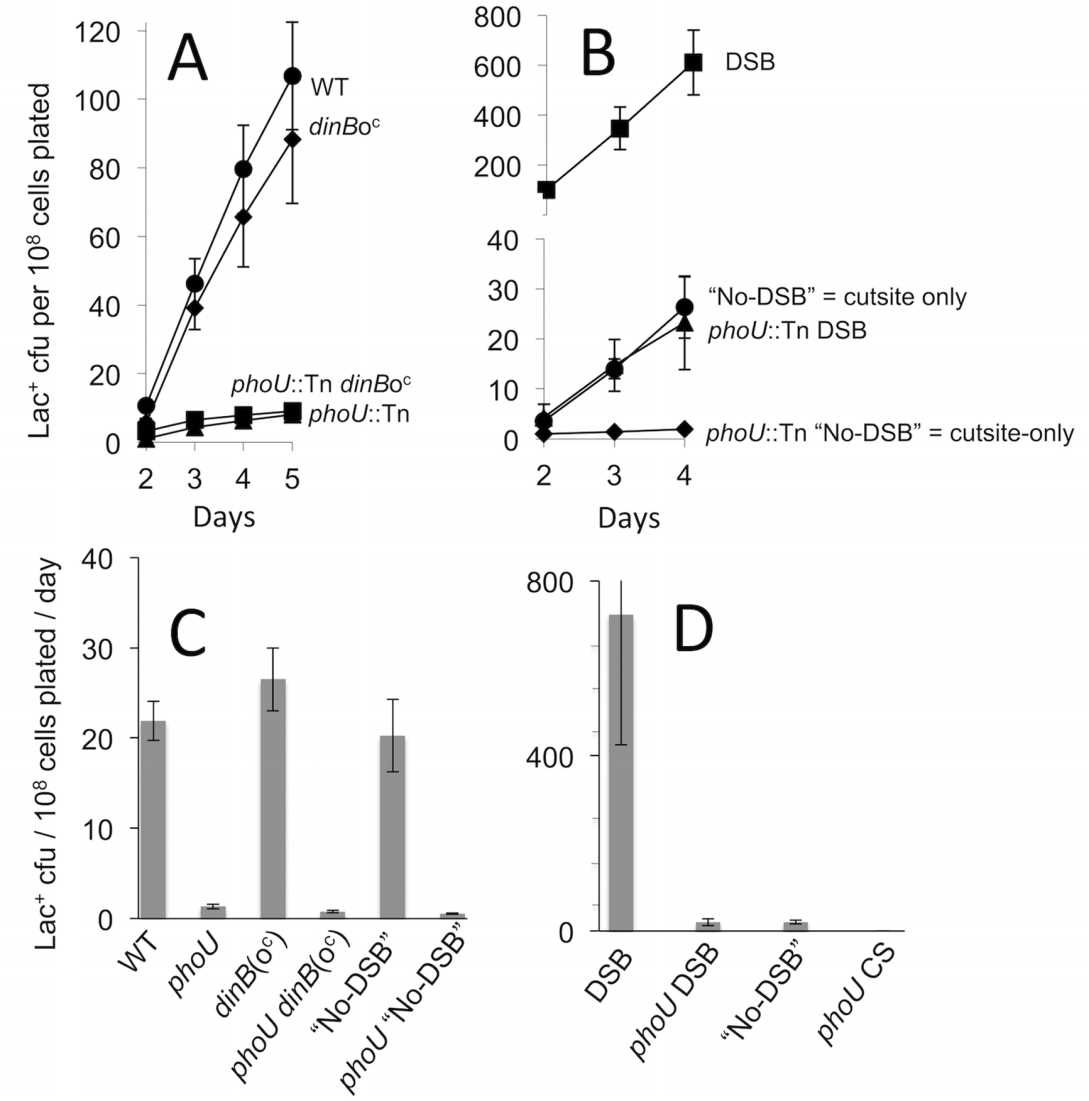
**PhoU is not substituted by SOS-induced levels of DinB (the SOS response) or by DSBs, the role of the σ^E^ response**, indicating that PhoU promotes MBR other than or in addition to by formation of DSBs, activation of the σ^E^ or SOS responses. (A, B) Representative experiments. (C, D) Multiple experiments. Lac^+^ mutation rates are Lac^+^ colonies/10^8^ cells /day from days 3–5 [46] (mean of 2–3 experiments ± range or SEM, respectively). The first set of isogenic strains carry a *dinB* operator-constitutive allele *dinB*(o^c^) [45], which produces SOS-induced levels of DinB protein at all times, and completely substitutes for a functional SOS response in MBR [45]. *dinB*(o^c^) does not substitute for functional PhoU, indicating that PhoU promotes mutagenesis other than or in addition to by promoting the SOS response. The isogenic strains in the right panel (and right side of the left panel) carry either inducible I-*Sce*I endonuclease and a cutsite near *lac* (DSB), or the cutsite-only (“No-DSB”), which has spontaneous DSBs but not additional DSBs induced by I-SceI. I-SceI-induced DSBs substitute for all components that contribute to spontaneous DSBs in the *lac* region: σ^E^ [17], TraI [15]; Mfd and RNA-DNA hybrids [36], but do not substitute for PhoU. Strains: WT, SMR4562; *phoU*, SMR4953; DSB, SMR6280; “No-DSB”, SMR6281; *phoU* DSB, SMR19235; *dinB*(o^c^), SMR17049; *phoU dinB*(o^c^), SMR20214. Rates were calculated from 3 separate experiments for *phoU*, wild-type and DSB strains, and error bars represent one SEM. For *dinB*(o^c^), error bars represent range calculated from two independent experiments.

We tested whether PhoU promotes mutagenesis via activation of the SOS DNA-damage response, the σ^E^, or σ^S^ responses, stress-sensing for and activation of which accounts for more than half of the functions in the 93-gene MBR network [18]. SOS is required for MBR [32,44], and though ~40 genes are induced in the SOS response, the induction of *dinB* alone is sufficient for stress-induced Lac reversion [45]. A *dinB* operator constitutive (o^c^) mutant that provides SOS-induced levels of DinB constitutively restores wild-type-like mutation rates to an SOS-non-inducible strain [45], bypassing the need for SOS induction. By contrast, we found that the *dinB*(o^c^) mutation did not restore mutagenesis in *phoU*83::Tn*10 dinB*(o^c^) cells (Fig 3A–3C). We conclude that PhoU functions in mutagenesis other than, or in addition to, by activation of the SOS response.

**Fig 4:**
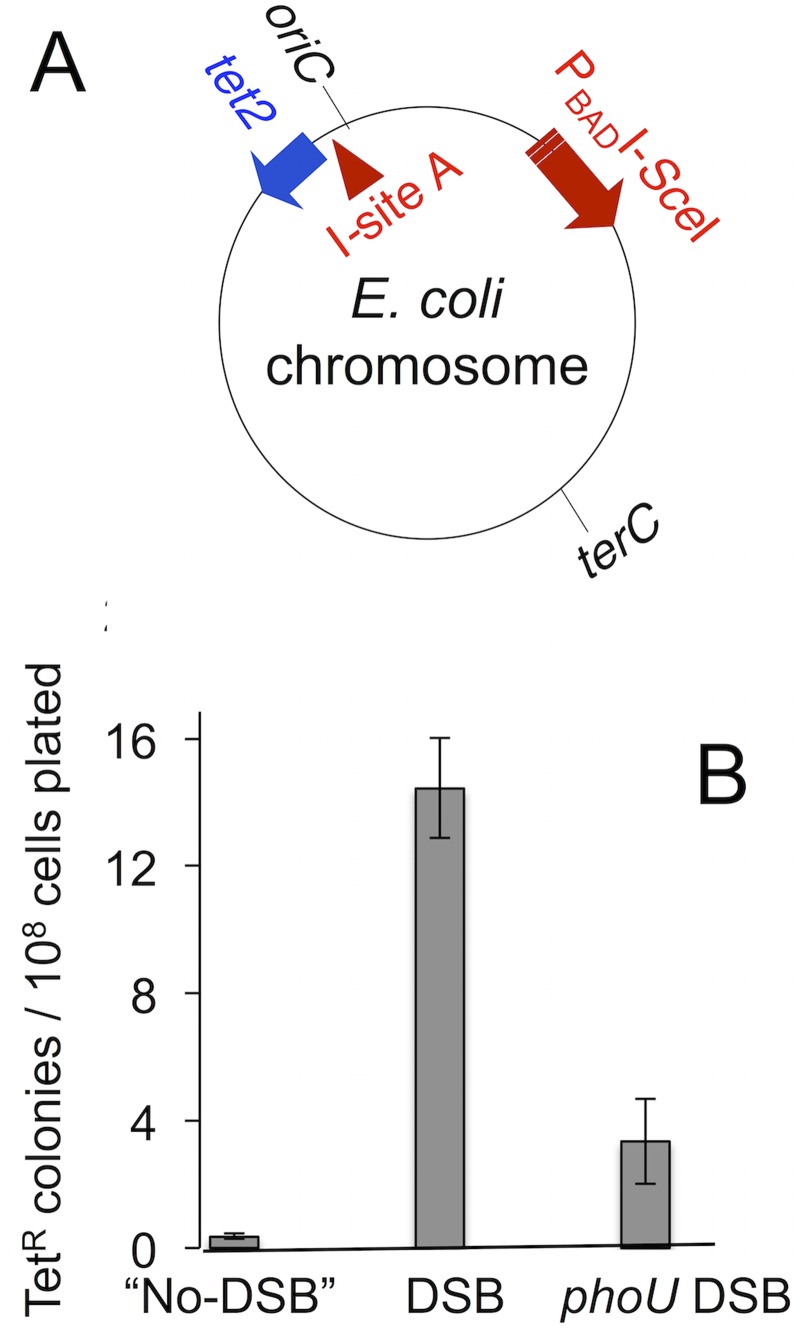
PhoU is required for MBR in the *E*. *coli* chromosome. (A) Diagram of relevant genetic elements in the *E*. *coli* chromosome. Experimental design of [16]. Cells expressing a chromosomal regulatable I-*Sce*I endonuclease gene and carrying a chromosomal cutsite near a *tet* +1bp frameshift allele are starved in liquid for 84 hours (with no tetracycline), rescued to rich medium then plated on rich tetracycline and no-drug plates to score tetracycline-resistant (TetR) mutant colonies. (B) PhoU is required for I-SceI-induced MBR under stress, and DSBs do not substitute for PhoU in mutagenesis. DSB strains have I-*Sce*I enzyme and cutsite and control “No-DSB” strains have I-*Sce*I cutsite only. Strains: “No-DSB”, SMR10865; DSB, SMR10866; *phoU* DSB, SMR20344. The DSB mutant frequency is 14.5 Tet^R^ mutants /10^8^ cells (1.5 x 10^-7^ TetR mutants per cell). Mean ± range of two independent experiments.

The σ^E^ response promotes MBR in the Lac assay mainly via its contribution to spontaneous DNA breakage, as evidenced by the finding that DSBs created by I-*Sce*I endonuclease near *lac* substitute for σ^E^ in mutagenesis [17]. I-*Sce*I cuts also substitute for requirements for DSB-promoting TraI single-strand endonuclease [15], RNA/DNA hybrids and Mfd RNA-polymerase translocase [36], but not for DSB-repair, SOS- or σ^S^-response functions, or error-prone DNA polymerases [2,15,16] (also [18]). We find that I-*Sce*I-generated DSBs did not restore mutagenesis-proficiency to *phoU83*::Tn10dCam cells in two assays. First, though I-*Sce*I cuts near *lac* increase overall Lac reversion ([15] and Fig 3B–3D), they did not relieve the strong *phoU83*::Tn10dCam mutagenesis defect (Fig 3B–3D). Second, we used the chromosomal Tet assay [16] in which I-*Sce*I cuts delivered near a chromosomal revertible *tet* +1bp frameshift allele in plasmid-free cells (Fig 4A) promote Tet reversion via σ^S^-, DinB-dependent MBR [16]. In the Tet assay as well, I-*Sce*I cuts did not substitute for PhoU^+^ (Fig 4B). We conclude that PhoU promotes mutagenesis by some mechanism other than, or in addition to, via promotion of spontaneous DSBs, and thus also other than, or in addition to, via activation of σ^E^.

### Post-translational σ^S^-activating mutations do not substitute for PhoU in mutagenesis

**Fig 5:**
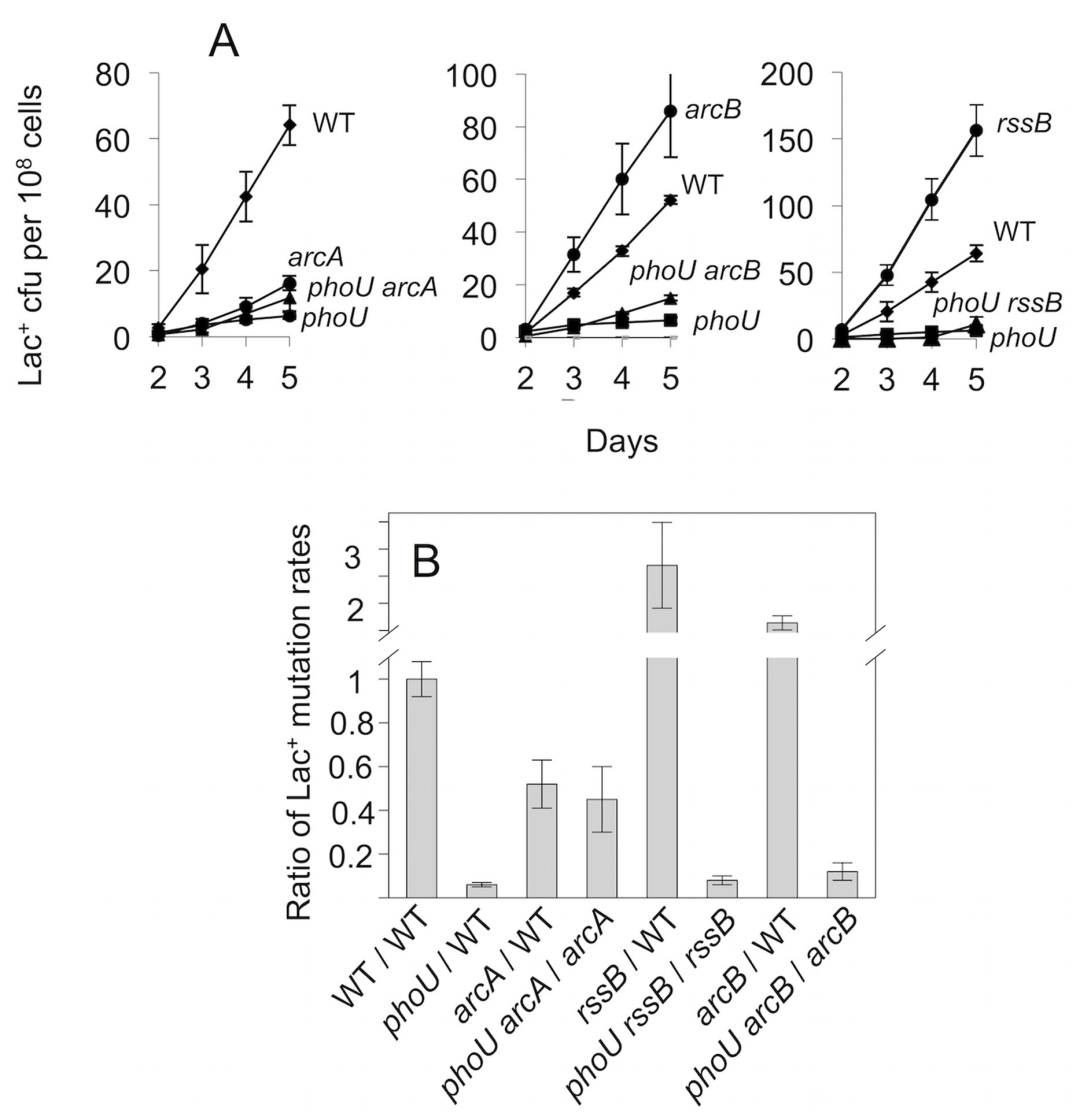
PhoU is not substituted by artificial upregulation of σ^S^ *via arcB* or *rssB* deletions, but is suppressed by deletion of *arcA*. (A) Representative data. (B) Data for three-four separate experiments (means ± SEM) showing ratios of the mutation rates (Lac^+^ colonies / 10^8^ cells plated / day between days 3–5 of experiments such as those in Fig 1A). The wild-type rate is 18.8 Lac^+^ colonies /10^8^ cells plated / day. Strains: WT, SMR4562; *rssB*, SMR12566; *arcA*, SMR12672; *arcB*, SMR12673; *phoU*. SMR4953; *phoU rssB*, SMR19248; *phoU arcA*, SMR19249; *phoU arcB*, SMR19250.

MBR requires the σ^S^ response [15,16,46] and 30% of the genes in the MBR network promote mutagenesis via sensing stress and signal transduction that activates the σ^S^ response [18]. Two ways to artificially up-regulate σ^S^ (and induce the σ^S^ response) are knock-out of RssB, which promotes σ^S^ proteolytic degradation, or ArcB, the histidine-kinase component of the Arc two-component signal-transduction system which negatively regulates σ^S^ post-translationally [47–49]. Δ*rssB* and Δ*arcB* mutations suppress many MBR defects resulting from insufficient σ^S^ [18]. By contrast, we find that neither Δ*arcB* nor Δ*rssB* restore mutagenesis to *phoU83*::Tn10dCam cells (Fig 5). The mutation rate was decreased dramatically by *phoU83*::Tn*10* in the *rssB* background (*phoU83*::Tn*10 rssB* compared with *rssB*, Fig 5) and the *arcB* background (*phoU83*::Tn*10 arcB* compared with *arcB*, Fig 5) suggesting that decreased mutagenesis may result from some mechanism other than, or in addition to, failure to induce the σ^S^ response. We cannot eliminate the possibility of a transcriptional upregulation of *rpoS* somehow caused by PhoU^+^, which might not be suppressed by mutations in *rssB* and *arcB*, which affect σ^S^ production post-translationally. However, many probable transcription-related defects in σ^S^ production were suppressed by *rssB* and *arcB* mutations [18], suggesting that PhoU does not promote mutagenesis via σ^S^ activation.

### Suppression of *phoU*::Tn*10*dCam mutation by *arcA*

Previously, a C-terminal transposon insertion in *phoU* was isolated in a screen for persister mutants, and conferred hyper-sensitivity to ampicillin [50]. Microarray analysis in the mutant showed overexpression of several energy-metabolism genes including *arcA*, the response regulator of the two-component Arc system [51]. In contrast with ArcB and RssB which negatively regulate σ^S^ post-translationally, ArcA represses transcription of σ^S^ [52]. Although mutations in each of the three cause net accumulation of σ^S^ in cells, *arcA* mutants are still slightly defective [2-fold reduced, [18] and Fig 5] in MBR, suggesting that the *arcA* role in mutagenesis is more complex than simple relief of σ^S^ repression [18]; that is, ArcA also promotes mutagenesis.

We find that deletion of *arcA* in a *phoU83*::Tn*10*dCam cell restores mutagenesis to levels similar to that of the *arcA* mutant (Fig 5A). That is, ArcA is epistatic to *phoU83*::Tn*10*dCam. *phoU83*::Tn*10*dCam decreases mutagenesis 13-times less in an *arcA* strain that in a wild-type strain. The *phoU83*::Tn*10*dCam *arcA*: *arcA* mutagenesis ratio is 13-fold higher than *phoU83*::Tn*10*dCam: WT (meaning there is 13-fold *less* depression of mutagenesis by *phoU83*::Tn*10*dCam in an *arcA* strain than in WT) indicating that *arcA* partially suppresses the *phoU* mutagenesis defect (Fig 5B).

## Discussion

The data presented show that PhoU and several proteins in phosphate regulation promote MBR in *E*. *coli* (Figs 2 and 4), in ways that do not correlate with their roles in phosphate regulation (Fig 2) indicating additional roles. PhoU^+^ does not promote mutagenesis mainly via activation of the SOS or σ^E^ responses (Figs 3 and 4) or via promotion of spontaneous DSBs that underlie MBR (Figs 3 and 4). The data suggest a possible PhoU mutagenic role partly via repression/antagonism of ArcA (Fig 5). The data also indicate that *phoU83*::Tn*10*dCam is a partial loss-of-function mutation that retains the (unknown) PhoU essential function, such that suppressor mutations are not required for viability, but lacks Pho-regulon repression (Table 2), and so may be a useful allele for studying PhoU functions in other pathways in which phoU mutations have phenotypes.

Although the roles of PhoU and the PhoBR regulon in MBR are complex, several points are clear. First, the ability to induce the Pho regulon is not required for MBR (a Δ*phoBR* strain, which lacks the PhoB transcriptional activator, is mutagenesis-proficient, Fig 2). This is not surprising given the high phosphate (repressing) conditions of the M9 minimal medium used in the mutation assay. Second, although some of the mutations examined lead to Pho regulon de-repression, inappropriate de-repression is not correlated with mutagenesis deficiency. For example, although *phoU83*::Tn*10*dCam is both mutagenesis-defective and de-repressed, other mutations that de-repress the regulon have no defect (*phoU35*, *phoR68*) or smaller defects [Δ*pstS*, Δ(*pstSCAB-phoU*)] (Fig 2A and 2B). Moreover, if the mild mutagenesis deficiencies of the Δ*pstS*, Δ(*pstSCAB-phoU*), and *phoU83*::Tn*10*dCam Δ*pstS* strains were caused by de-repression of the regulon, then introduction of the Δ*phoBR* deletion, which makes the regulon uninducible, should restore full mutagenesis proficiency, and this was not observed for the first two strains (though it was for third, Fig 2B).

The role of PhoU in MBR is difficult to model because our data do not correlate perfectly with the two functions, one known and one hypothesized, of PhoU. PhoU is required for repression of the Pho regulon in high phosphate, and also to prevent a PstSCAB-dependent inhibition of growth [20,23,39]. As just discussed, de-repression of the regulon associated with *phoU* and *pstSCAB* mutations does not inhibit mutagenesis per se. *phoU83*::Tn*10*dCam confers a growth defect (Tables 2 and 3), and mutagenesis-deficiency. Normal growth is restored (as expected [20]) by introducing mutations that block PstSCAB activity [Δ*pstS*, Δ(*pstSCAB-phoU*)] or prevent its induction (Δ*phoBR*), but mutagenesis-proficiency is only partially restored (Fig 2B and Results). Thus, we suggest that the strong phenotype of *phoU83*::Tn*10*dCam results from two deficiencies: a failure to block PstSCAB-mediated growth-inhibition, and lack of another, unknown, function of PhoU (not its regulon-repressing function). That the other *phoU* mutant strains all form colonies efficiently under selective conditions (Table 3) is consistent with the other PhoU function being necessary for MBR. A possible reason for the mutagenesis deficiency might be the level of polyphosphate within the cell. MBR is depressed by polyphosphate levels that are either too high or too low [53], and PhoU mutants have high levels of polyphosphate, whereas mutations in the PstSCAB transport genes lead to low polyphosphate levels [54,55]. Thus, mis-regulated polyphosphate levels are a plausible possible cause of part of the *phoU* mutagenesis-deficiency. Further experiments are required to test this hypothesis.

Our results suggest additional complexities to PhoU function. In addition to its separate functions in repressing the Pho regulon, preventing a *pstSCAB*-dependent growth defect (separable genetically by the *phoU35* mutation, [20,23]), and its positive role in mutagenesis (here), PhoU can also inhibit mutagenesis. Inhibition is manifested as a strong PhoU-dependent mutagenesis deficiency in *pstS phoBR* cells, in which *pstS*-dependent effects are not a factor and the Pho regulon is uninducible (Fig 2B). The presence of either *pstS* ^+^ or *phoBR* ^+^, or mutation of *phoU* can alleviate this defect. Thus, it appears that the PhoU^+^ function in mutagenesis must be constrained by either *pstS* ^+^ or *phoBR* ^+^ to achieve wild-type levels of MBR. This could reflect direct interactions with PstS(CAB) or PhoB or PhoR, all of which interact genetically to regulate the regulon, and are proposed to form a repressing complex at the membrane [20,21] (Fig 1), regulation of polyphosphate levels, or other. How PhoU both promotes and hinders mutagenesis, and what the relationship is between those two functions, remains to be determined.

The Pho regulon is required for survival in adverse conditions and so, not surprisingly, up-regulates expression of σ^S^ [56,57]. *pstS* mutation increases σ^S^ levels *phoBR*-dependently in exponentially growing cells, but not in stationary-phase cells, apparently mediated via a small regulatory RNA [56]. However, the effects of Pho mutations on σ^S^ expression, manifested only in exponentially growing cells, are not congruent with their effects on MBR, suggesting that Pho does not affect mutagenesis via σ^S^ up-regulation. Supporting a PhoU role other than in σ^S^-response activation, neither *rssB* nor *arcB* deletion, both of which increase σ^S^ levels, restored mutagenesis to *phoU83*::Tn*10*dCam cells (Fig 5), whereas restoration of mutagenesis by Δ*rssB* and Δ*arcB* was seen for several σ^S^-activator mutations [18]. Suppression of the *phoU* phenotype by Δ*arcA* (Fig 5) coupled with known *arcA* repression by PhoU [50] suggests that a gene repressed by ArcA may be required for MBR, and that the PhoU repression of *arcA* might underlie its role in mutagenesis.
